# The Role of Catalyst Promotive Additives and Temperature in the Hydroisodewaxing Process

**DOI:** 10.3390/molecules28227598

**Published:** 2023-11-15

**Authors:** Kamilla Abdildina, Gulzira Vassilina, Albina Abdrassilova, Ivan A. Klassen, Raigul Orynbassar, Fatima Kanapiyeva

**Affiliations:** 1 Department of Physical Chemistry, Catalysis and Petrochemistry, Al-Farabi Kazakh National University, Almaty 050040, Kazakhstan; v_gulzira@mail.ru (G.V.); albina06.07@mail.ru (A.A.); merkrunner123@gmail.com (I.A.K.); fatima31@mail.ru (F.K.); 2 Department of Chemistry and Chemical Technology, K. Zhubanov Aktobe Regional University, Aktobe 030000, Kazakhstan; raihan_06_79@mail.ru

**Keywords:** diesel fuel, hydroisodewaxing, isodewaxing, hydroisomerization, mesoporous aluminosilicate, bifunctional catalyst, diesel fuel performance characteristics, Ni or Mo catalysts, cold filter plugging point, CFPP, flash point in a closed cup, pour point

## Abstract

One of the valuable fractions of paraffinic oils is the diesel fraction, which can be used as a commercial fuel. However, the high content of alkanes of normal structure (~10–40%) in the diesel fraction leads to a deterioration in the performance characteristics of the fuel and, as a result, the inability to use the diesel fraction without additional processing in the cold season at lower temperatures, which is critical for many regions with cold winters. The process of catalytic dewaxing is one of the most promising ways to improve the low-temperature characteristics of diesel fractions. This work is devoted to studying the activity of promoted Ni, Mo, and Ni-Mo catalysts based on mesoporous aluminosilicate and pre-activated bentonite in dewaxing diesel fractions. The effect of the nature and content of promoting additives on the activity of bifunctional catalysts in the process of hydroisodewaxing of diesel fraction in a flow-type reactor in the temperature range of 260–340 °C, pressure of 2 MPa and feed space velocity of 1 h^–1^ was studied. It is shown that the synthesized bifunctional catalysts based on mesoporous aluminosilicate and pre-activated bentonite from the Tagan field (Ni/MAS-H-bentonite, Mo/MAS-H-bentonite, and Ni-Mo/MAS-H-bentonite) have the necessary balance of Lewis and Bronsted acid centers strengths. It allows them to selectively conduct the hydroisodewaxing process. It has been established that the use of the synthesized 5% Ni-1% Mo/MAS-H-bentonite bifunctional catalyst in the diesel fractions hydroisodewaxing process under optimal process conditions makes it possible to obtain diesel fuel with low-temperature characteristics that meet the requirements for cold climate fuels: cold filter plugging point (CFPP)—minus 33 °C, flash point in a closed cup—39 °C and pour point—minus 36 °C.

## 1. Introduction

Diesel fuel is a yellow liquid viscous substance used in diesel engines. It is usually produced by distillation of oil and obtaining kerosene-gas oil fractions. The name “diesel fuel” comes from the name of German engineer Rudolf Diesel. In 1892, Diesel applied for a patent for his invention: “a new rational heat engine” [1].

According to the Ministry of Energy of the Republic of Kazakhstan, diesel fuel consumption in 2023 is projected to be 5.8 million tons, compared with a production of 5.1 million tons. In 2022, this ratio was 5.6 and 5.3 million tons, respectively. However, the growth in consumption is not associated with the main sectors of the economy, such as the mining and metallurgical complex (MMC) and the railway, but is growing due to transit vehicles and “gray” exports to neighboring countries due to the cheap cost of fuel [2]. The increasing demand for diesel fuel consumption challenges scientists to improve processing and production, improve environmental performance, develop new technologies, and research aimed at more efficient and sustainable use of this type of fuel. However, the high content of normal alkanes (~10–40%) in the composition of the diesel fraction leads to a deterioration in the performance of the fuel and, as a result, the inability to use the diesel fraction without additional processing in the cold season, which is critical for many regions of Kazakhstan. Indicators such as pour point, freezing point, viscosity, and viscosity index are significantly improved when side chains are introduced along linear hydrocarbon chains [3,4].

Until the end of the 20th century, the main technological process that made it possible to reduce the pour point of raw materials was catalytic dewaxing, based mainly on hydrocracking reactions. However, lowering the pour point due to the selective hydrocracking of feedstock n-paraffins to light hydrocarbons leads to significant losses of the target product. Isodewaxing, in its turn, improves the low-temperature characteristics of crude oil. The process of isodewaxing is based on the hydroisomerization reactions of n-paraffins because the isoparaffins obtained in the course of the reaction remain in the composition of the target product [5]. Usually, the temperature of the hydroisomerization process varies from 280 to 350 °C, achieving a relatively high selectivity for the desired products [6].

Bifunctional catalysts are widely used for the hydroisomerization of n-alkanes. The presence of metal and acid sites in them makes it possible to provide the hydrogenation/dehydrogenation function for the saturation/generation of alkene intermediates and the acidic function for skeletal rearrangement according to the carbenium ion mechanism [7,8,9]. The key to making this combination an excellent catalyst to achieve the aforementioned goal is the balance of acidic and metallic properties of the catalysts [10].

Catalytic systems of the isodeparaffinization process primarily utilize expensive metals such as platinum and palladium [11,12]. However, since these metals impart dehydrating properties to catalysts, they are not resistant to the toxic effects of contact poisons present in the diesel fraction. To address this issue, it is proposed to employ catalysts promoted by metals such as Ni and Mo; this is attributed to the fact that catalysts based on these metals exhibit high hydrogenating activity even in the presence of significant sulfur content in the raw material [13,14,15]. On the other hand, the use of this type of metal significantly reduces production costs and increases the volume of fuel produced. The choice of nickel, molybdenum, and the bimetallic Ni-Mo system as promoting components was due to the fact that nickel can compete with platinum- and palladium-based catalysts in its hydrogenation activity, as well as give catalysts dehydrogenating properties. At the same time, molybdenum demonstrates high resistance to poisoning by sulfur- and nitrogen-containing compounds [16,17]. The combination of nickel and molybdenum gives their mixture the ability to simultaneously carry out both homolytic and heterolytic reactions and gain resistance to the toxic effects of the sulfur and nitrogen compounds contained in petroleum feedstocks [18].

In this regard, the purpose of this work is to improve the performance characteristics of diesel fuels by hydrodewaxing in the presence of nickel-, molybdenum- and nickel-molybdenum-containing bifunctional catalysts based on mesoporous aluminosilicate and pre-activated bentonite.

To achieve the goal of the work, bifunctional catalysts (Ni/MAS-H-bentonite, Mo/MAS-H-bentonite, and Ni-Mo/MAS-H-bentonite) were synthesized, their physicochemical characteristics and catalytic activity in the process of hydrodewaxing of diesel fractions were studied.

The novelty of this work lies in the development of new catalysts based on mesoporous aluminosilicate and activated bentonite promoted by Ni and Mo metals for the dewaxing process of diesel fuel.

## 2. Results and Discussion

### 2.1. Physicochemical Characteristics of Bifunctional Catalysts

Synthesized mesoporous aluminosilicate (MAS) samples became one of the bifunctional composite support components and the bentonite of the Tagan field (East Kazakhstan region, Kazakhstan). The use of preliminarily activated bentonite as a second component for the catalyst support (MAS-H-bentonite) was conditioned by its accessibility, cheapness, developed specific surface area, durability, and thermal stability [19,20].

The authors studied and presented the physicochemical characteristics of mesoporous aluminosilicate and some catalysts [21]. In the current article, the additional results are presented.

Texture characteristics are critical to the final catalytic properties of the synthesized catalysts.

**Figure 1:**
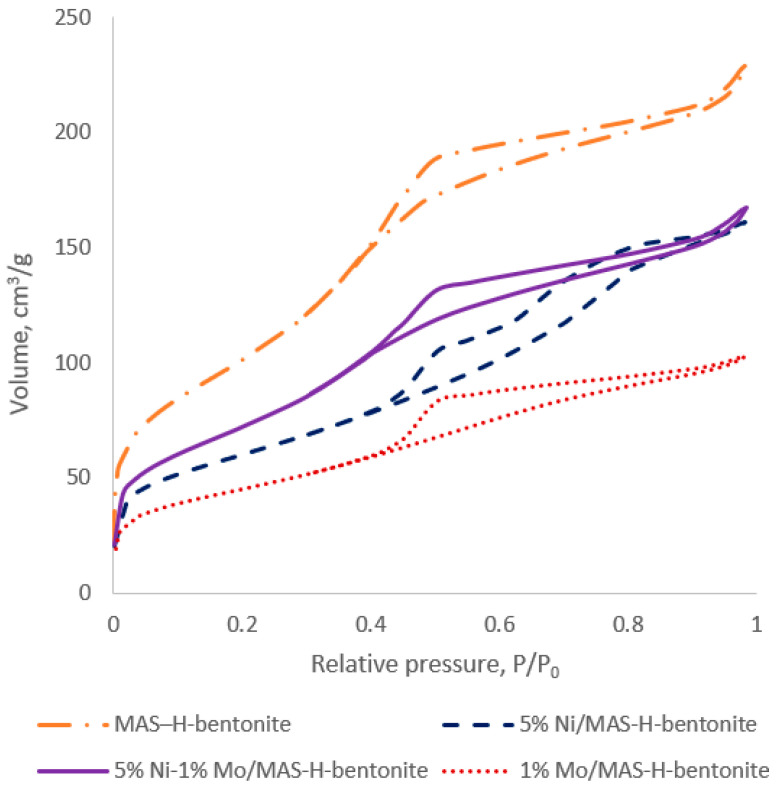
Nitrogen adsorption–desorption isotherms of the synthesized catalysts.

**Figure 2:**
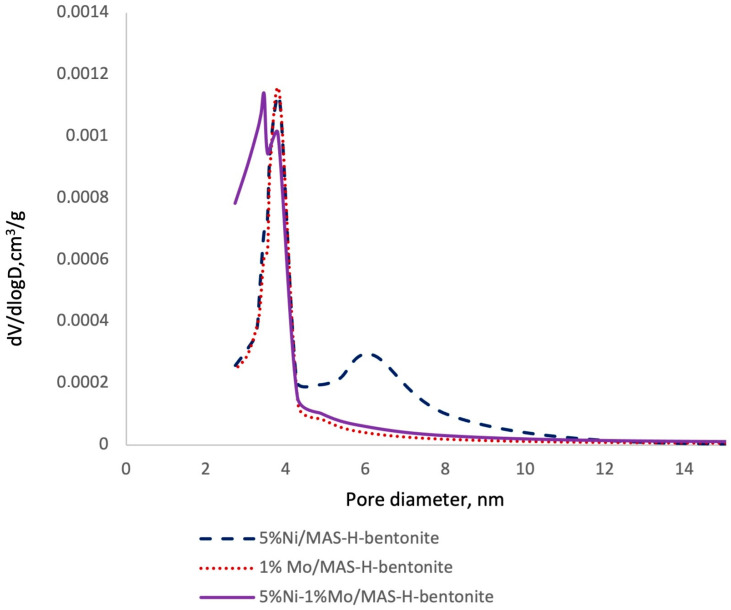
Synthesized catalysts pore sizes distribution.

The porous structure of the synthesized catalysts on the basis of mesoporous aluminosilicate is represented in Figure 1 and Figure 2 and Table 1.

The bifunctional catalyst isotherm pattern is similar to mesoporous aluminosilicates [18], indicating no carrier porosity damage after metal promotion. All obtained isotherms belong to the IV type of IUPAC classification, i.e., they have a more pronounced hysteresis loop closer to H4. They contain a hysteresis loop at relative pressures higher than P/P_0_ = 0.4, which is typical for mesoporous materials [22]. The presence of the hysteresis loop in the low-pressure range can be explained via capillary condensation in mesopores. The IV-type isotherms demonstrate a narrow pore size distribution in the 2–50 nm range. At the same time, the type H4 hysteresis is associated with tight slit-like pores [23].

The pore size distribution is presented in Figure 2 and was determined via the BJH method. According to the BJH analysis, the catalysts’ average pore size ranges from 1.67 to 3.8 nm. It was found that all samples show bimodal pore size distribution characteristics with maxima in the 2.9–6.9 nm range, which corresponds to mesopores. [24].

The specific surface area, pore volume, and average diameter of the synthesized materials are shown in Table 1.

According to the data in Table 1, there was a decrease in the specific surface area and pore volume of the catalysts promoted by nickel and molybdenum compared to MAS-H-bentonite’s specific surface area. Possibly, this is due to a partial block of the aluminosilicate pore system by the promoting additives. The specific surface area and the decrease in pore volume are the main indicators of metal attachment to carrier pores [25].

**Figure 3:**
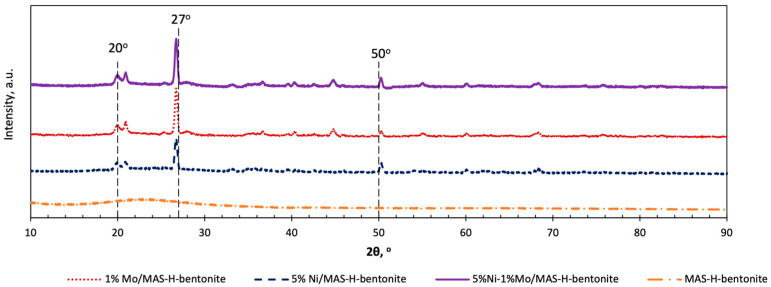
The wide angle-scattering pattern of MAS-H-bentonite and catalysts based on mesoporous aluminosilicates and pre-activated bentonite.

Further approvals of a final structure formation were obtained by measurements of X-ray diffraction on the promoted catalysts based on MAS and H-bentonite. An X-ray phase analysis of the supported nickel and molybdenum catalysts gives useful information about the presence or absence of Ni- and Mo-phases. X-ray diffraction patterns of the catalysts containing nickel and molybdenum in the 2θ range 10–90 are presented in Figure 3.

The wide angle-scattering pattern of MAS-H-bentonite shows one wide hump in the region 2θ = 20–30°, which indicates the absence of any crystalline phases. In contrast, diffraction peaks appear in the XRD patterns of Ni and/or Mo containing samples at 2θ − 20°, 27° and 50°. As the angle positions of these peaks do not depend on the kind of promoting metal, one can conclude that the incorporation of Ni and/or Mo oxo-species in the support matrix provokes a better ordering of its crystal structure [26,27].

Another important characteristic of bifunctional catalysts is the strength of acid sites. The best isomer selectivity is provided by carrier acidity, which is determined by the optimal ratio of strong Brønsted acid and weak Lewis acid sites [28].

**Figure 4:**
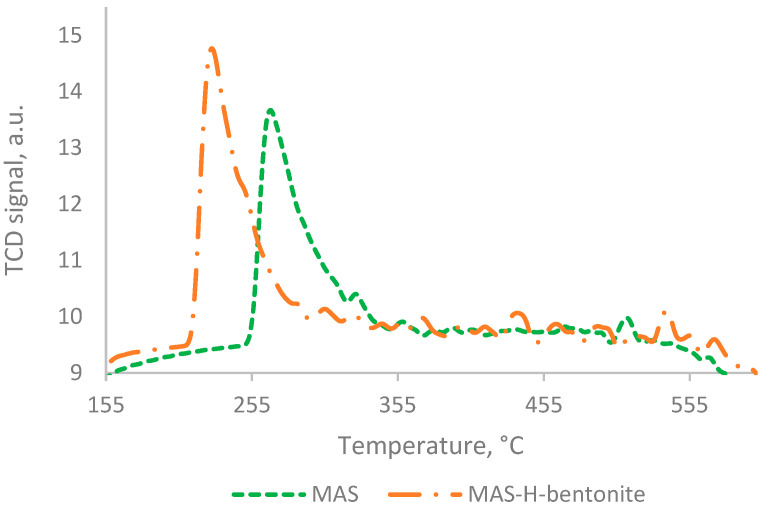
TPD—NH_3_ curves of mesoporous aluminosilicate and unpromoted composite based on mesoporous aluminosilicate.

**Figure 5:**
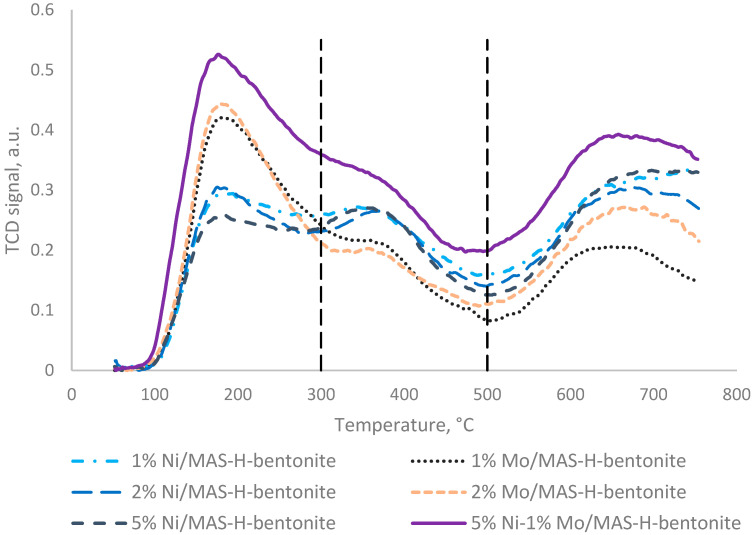
TPD—NH_3_ curves of synthesized bifunctional catalysts based on mesoporous aluminosilicates and pre-activated bentonite.

The TPD profiles of NH_3_ adsorbed at 100 °C on the sample precursors are presented in Figure 4 and Figure 5. The concentration of various acid sites, expressed in µmol of desorbed NH_3_ per 1 g of catalyst, is presented in Table 2 and Table 3.

According to Figure 4 and Table 2, for the mesoporous aluminosilicate and MAS-H-bentonite samples, the ammonia desorption curves have the same shape, i.e., the shape with one thermal desorption peak. However, these samples differ in strength and concentration of acid sites. So, for instance, a sample of synthesized mesoporous aluminosilicate has approximately 98% weak acid sites (1144 µmol/g) and 2% medium-strength acid sites (27 µmol/g). Mixing the mesoporous aluminosilicate with the activated bentonite leads to a shift in the ammonia desorption peak towards lower temperatures. However, the area of the ammonia desorption curve becomes bigger.

The data comparison in Figure 4 and Figure 5 and Table 2 and Table 3 shows that in contrast to the composite based on mesoporous aluminosilicate and H-bentonite, the thermal desorption curves of NH_3_ for the samples promoted with nickel and molybdenum have a different character. While the strength of acid sites increases, a decrease in their total concentration is also observed. Perhaps this is conditioned by blocking acid sites by the promoting additive.

On the TPD—NH_3_ curves of nickel-promoted samples, three thermal desorption peaks are observed with clearly defined maximums at low, medium, and high temperatures. The first low-temperature peak (T_max_ = 180–185 °C) is usually attributed to the desorption of ammonia adsorbed on the weak acid sites; the second peak (T_max_ = 350–365 °C) is usually attributed to the desorption of ammonia adsorbed on the medium acid sites [26,27,28,29,30,31]. The high-temperature peak above 500 °C corresponds to the strong acid sites. According to the data obtained and the thermal desorption of ammonia (Table 3), on the surface of the synthesized samples promoted with nickel, there are the weak acid centers—44–57%; the medium acid centers—35–41%; and only 7–18% can be attributed to the strong acid centers.

The thermal desorption curves of the catalyst samples promoted with molybdenum, in contrast to the samples promoted with nickel, have two maximums at low and high temperatures and a shoulder at medium temperatures. The samples are mainly characterized by weak acid sites of 64–68%. An increase in the strength and number of acid sites on the carrier’s surface with the introduction of a molybdenum source can be explained by an increase in the number of coordinately unsaturated Al^3+^ ions due to the tendency of Mo^6+^ ions to “pull” oxygen ions onto themselves [32].

It should be noted that with an increase in the content of both nickel and molybdenum in the catalyst, the total concentration of acid sites increased from 446 to 521 and from 620 to 684 °C, respectively; this happens mainly due to the strong acid sites and is tied up to the migration of nickel and molybdenum into the carrier channels and its interaction with the strong acid sites [33].

With the joint promotion of catalysts based on mesoporous aluminosilicate and pre-activated bentonite, an increase in the concentration of low- and high-temperature acid sites is observed.

Thus, from the TPD-NH_3_ results, it has been shown that the nature and content of promoting catalysts’ additives affect the concentration and strength of the acid sites. The 5% Ni-1% Mo/MAS-H-bentonite sample has a greater number of acid sites available for the chemisorption of NH_3_ than the samples of catalysts promoted only by nickel and molybdenum.

**Figure 6:**
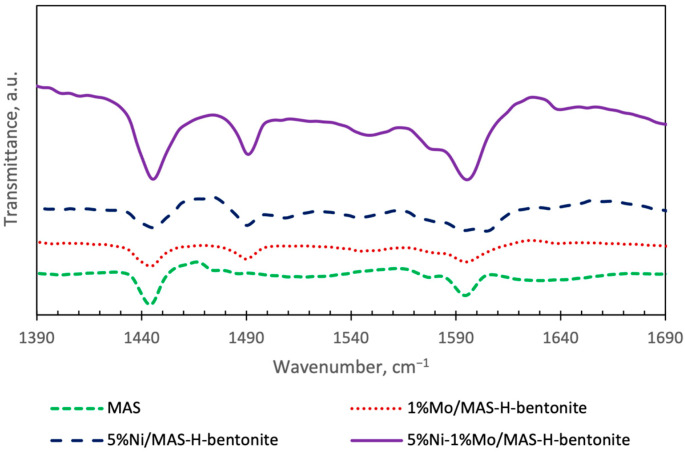
Fourier transform IR spectra of diffuse reflectance of mesoporous aluminosilicate and bifunctional catalysts.

To determine the relative strength of Brønsted and Lewis acid sites on the surface of mesoporous aluminosilicates and bifunctional catalysts based on them, the IR spectra of adsorbed pyridine samples were analyzed (Figure 6).

The absorption bands are fixed at 1445–1447, 1490, and 1595 cm^–1^ on the studied samples, as in the case of mesoporous aluminosilicates. The absorption bands at 1445 and 1595 cm^–1^ are explained by the presence of hydrogen-bonded pyridine adsorbed on Lewis acid sites. A Strong Lewis bond is established with pyridine at 1445–1447 cm^−1,^ and a weak Lewis bond is established with pyridine at 1595 cm^−1^ [34]. The band observed at about 1490 cm^–1^ is associated with the adsorption of pyridine on both Lewis and Brønsted acid sites. The appearance of a band at 1550 cm^−1^ in the IR spectrum of Ni-Mo/MAS-H-bentonite catalyst is explained by the presence of hydrogen-bonded pyridine adsorbed on Brønsted acid sites. It can be seen from the diffuse reflectance IR spectra that the Ni-Mo/MAS-H-bentonite catalyst sample has stronger Lewis and Brønsted sites and high acidity compared to monometallic catalysts, which matches well with the TPD-NH_3_ data.

Thus, based on the data obtained, it can be argued that catalysts based on mesoporous aluminosilicate promoted with nickel and molybdenum differ in strength and concentration of acid sites. The strongest acid sites are characteristic of the catalyst sample based on mesoporous aluminosilicate promoted simultaneously with nickel and molybdenum ions.

**Figure 7:**
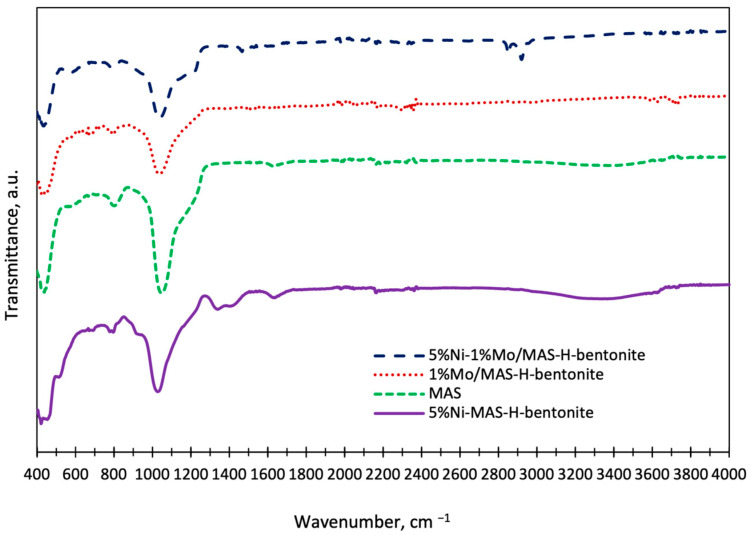
Fourier IR spectra of synthesized mesoporous aluminosilicate and bifunctional catalysts based on it.

FT-IR spectroscopy was used to study the retention of crystalline ordering and metal–carrier interaction in the catalysts. Figure 7 shows the IR-Fourier spectra of bifunctional catalysts in the wavenumber range of 400–4000 cm^−1^.

Spectra obtained by FT-IR spectroscopy for mono- and bimetallic systems containing MAS and pre-activated bentonite demonstrate the appearance of the same absorption bands at 450, 800, 930, 1059, 1350, and 1620 cm^−1^.

In the IR spectrums of bifunctional catalysts, the peak at 1059 cm^−1^ was attributed to the asymmetric stretching of Si-O-Si and Al-O-Al and the peak at 800 cm^−1^ was attributed to the stretching of SiO_4_^4−^ tetrahedral structural units. The peak at 930 cm^−1^ in the IR spectra of Ni and Ni-Mo promoted catalysts was attributed to Si-O stretching vibrations with neighboring metal ions. This peak was used to characterize the incorporation of metal ions into the Si-O-Me silica framework [19]. Moreover, according to the shape of the shoulder at 930 cm^−1,^ which is combined with the peak at 1059 cm^−1^, it can be said that the metal-support interaction is very strong, according to [35]. The evidence of the Si-O-Ni formation by the change in the vibrations of the Si-OH groups in the band at 961 cm^−1^ was also presented in the next work [36].

In the IR spectra, the range at wavenumbers is less than 500, and the peak at 1350 cm^−1^ is associated with Si-O-Si vibrations. The presence of a peak at 1620 cm^−1^ associated with the O-H bond is explained by the presence of particles of aluminum/silicon hydroxide and trapped water.

**Figure 8:**
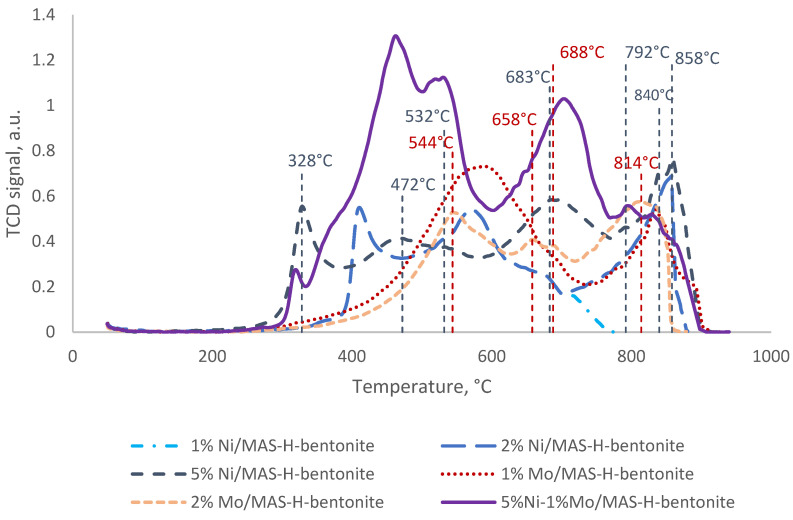
Curves of the temperature-programmed hydrogen reduction of bifunctional catalysts.

The temperature-programmed hydrogen reduction method studied the interaction of nickel and molybdenum with supports and between both metals. TPR graphs of the obtained oxide precursors, depending on the nickel and molybdenum content in the range of 1–5 and 1–2 wt.%, respectively, are shown in Figure 8.

On the curves of the catalysts promoted with 1 wt.% nickel, six peaks are observed: 328, 472, 532, 683, 840, and 858 °C and a shoulder at 792 °C (Table 4). With the increase of nickel content in the catalyst, the low-temperature peak shifts toward higher temperatures. The peak at about 328 °C corresponds to the reduction of free NiO in the catalyst support [37,38]. This reduction peak corresponds to the reduction of NiO particles, which have minimal or no interaction with the support. The reduction peaks from 450 °C to about 800 °C may be due to the strong interaction between NiO and the catalyst support. The peak at 532 °C refers to the reduction of Ni^2+^ to NiO [39] and the reduction of nickel oxide monolayer particles on the catalyst surface [40,41]. High reduction temperatures (683 and 792 °C) may indicate NiAl_2_O_4_, a hard-to-recover spinel [42,43]. The nickel-promoted sample also had reduction peaks at 840 and 858 °C. These high reduction temperatures are associated with the reduction of Ni^2+^ to Ni interacting strongly with the alumina support due to the metal alloy effect [37,44].

In the case of the MAS-H-bentonite catalyst promoted with 1 wt.% Mo, four peaks were observed on the TPR curve: at 544, 658, 688, and 814 °C, and in the case of the sample promoted with 2 wt.% Mo, two peaks at 589 and 834 °C were observed.

The peak at 544 °C is conditioned by the reduction of polymeric octahedral forms of molybdenum Mo^6+^ → Mo^4+^ [45]. Other reduction peaks from 589 to 688 °C may be associated with the reduction of molybdenum oxide (Mo^4+^) to metallic molybdenum (Mo^0^) [25,46]. High-temperature peaks at 814 and 834 °C include a deep reduction of all forms of Mo, including highly dispersed tetrahedral forms of Mo [47].

Thus, both octahedral and tetrahedral forms of molybdenum coexist on the surface of a 2% Mo/MAS-H-bentonite catalyst, as observed in the IR spectra.

It is known [48] that MoO_3_ without support is reduced in two stages:

MoO_3_ + H_2_ → MoO_2_ + H_2_O

MoO_2_ + 2H_2_ → Mo + 2H_2_O.

However, this definition is not unambiguous because different types of molybdenum interact with the surface of the two substrates to different degrees, which is reflected in the low and high-temperature peaks.

It is also known [25,49] that the tetrahedral forms of molybdenum are difficult to reduce, while the octahedral forms and other polyhedral forms are easily reduced. Based on the above, the low-temperature peak can be assigned to the octahedral forms of molybdenum, and the high-temperature peak can be assigned to the tetrahedral forms.

Co-promotion of composite based on MAS and H-bentonite shifts all reduction peaks to lower temperatures. Peaks were observed at 317, 460, 529, 702, 792, and 827 °C; this means the reducing ability of molybdenum and nickel is increased. The peak at 460 °C is associated with the reduction of the NiMoO_4_ phase [50], which corresponds to the wide-angle scattering data.

Thus, the synthesized bifunctional catalysts based on mesoporous aluminosilicate and pre-activated bentonite were characterized by low-temperature N_2_ adsorption/desorption, X-ray diffraction, TPD-NH_3_, diffuse reflectance FT-IR spectroscopy, FT-IR spectroscopy and TPR-H_2_. The distribution of pore sizes and the hysteresis loop of nitrogen adsorption/desorption isotherms of the catalysts confirm the preservation of the hexagonal mesoporous structure even after the introduction of nickel and molybdenum. The X-ray diffraction pattern demonstrates the formation of crystalline phases of nickel and molybdenum. According to TPD-NH_3_ and IR-Fourier diffuse reflectance spectroscopy, it was shown that all bifunctional catalysts are characterized by weak, medium, and strong acid sites, and the catalyst co-promoted with nickel and molybdenum possesses higher acidity. The reduction ability of the promoting additives of 5% Ni-1% Mo/MAS-H-bentonite catalyst, according to the results of TPR-H_2_, increases.

### 2.2. Characteristics of Diesel Oil Fractions before the Experiments

One of the most important criteria for the operational efficiency of diesel fuels used in cold climatic zones is good low-temperature properties, the values of which determine the operation of the engine power system at negative ambient temperatures and under fuel storage conditions. Low-temperature properties are determined primarily by the content of unbranched paraffins and are characterized by three indicators: pour point, flash point in a closed cup, and cold filter plugging point (CFPP).

The object of study in testing the synthesized samples of catalysts was straight-run hydrotreated diesel fraction. The physicochemical parameters of the diesel fraction are shown in Table 5.

The group hydrocarbon composition was determined using the chromate-mass spectrometry method to obtain more detailed information about the composition of diesel fractions. This method provides more detailed information about the composition of diesel fractions, as it is able to separate them into different groups of hydrocarbon compounds, which helps to better understand the chemical characteristics of diesel fractions and their potential impact on the environment and diesel engine performance. The group hydrocarbon composition of the studied diesel fraction is presented in Table 6.

Analysis of the group hydrocarbon composition of the investigated diesel fraction shows that the original diesel fraction contains a significant amount of n-paraffins (18.15 wt.%), which determines the temperature properties of the diesel fraction. The composition of paraffinic hydrocarbons is characterized by hydrocarbons from C_10_ to C_27_. Obviously, when the fuel has a lower n-alkane concentration and a higher iso-alkane content, the cloud point (the temperature at which crystals begin to form) and the maximum filterability of the fuel are reduced, indicating an improvement in performance at lower temperatures. In addition, the raw material contains more than 20 wt.% monoaromatic hydrocarbons. The diesel fraction also contains small amounts of polycyclic aromatic compounds and compounds with heteroatoms.

### 2.3. Investigation of the Activity of the Synthesized Catalysts in the Process of Isodewaxing the Diesel Fraction

The developed bifunctional catalysts were tested in the diesel fraction isodewaxing process. The bifunctionality of the synthesized catalysts is determined by the presence of metal and acid sites in them, which makes it possible to provide the hydrogenation/dehydrogenation function for saturation/generation of alkene intermediates, and the acidic function, skeletal rearrangement by the carbenium ion mechanism. Nickel, molybdenum, and nickel-molybdenum systems were used as the hydro/dehydrogenating components, and a mixture of mesoporous aluminosilicate and pre-activated bentonite was used as the acid component.

The catalytic activity of the synthesized bifunctional catalysts based on mesoporous aluminosilicates was studied in a flow-type unit with a fixed catalyst bed. Experimental studies were carried out under the following conditions: in the temperature range 260–340 °C, 2 MPa, H_2_; the raw material feed rate was 1 h^−1^, and the volume ratio H_2_:raw material = 100. To determine the yield of the diesel fraction, the products obtained from hydroisodewaxing were stabilized to remove low-boiling products. The results of catalytic hydroisodewaxing of diesel fuels are presented in Table 7 and Table 8.

Analysis of the data in Table 7 shows that the temperature properties of the obtained products depend on the amount of molybdenum in the composition of the Mo/MAS-H-bentonite catalyst. In both cases, with an increase in the process temperature from 260 to 340 °C, an improvement in performance is observed: the pour point of the products decreases from −26 to −41 °C, and the CFPP also decreases from −14 to −42 °C, possibly due to the hydrocracking products of long-chain n-paraffins, which tend to aggregate in diesel fuel and clog fuel filters. However, an increase in the process temperature decreases the diesel fraction yield; this is especially noticeable in the products obtained by the hydrodewaxing process of diesel fraction on the 2% Mo-containing catalyst based on mesoporous aluminosilicate. The decrease in the yield of the target product can be explained by an increase in the proportion of secondary hydrocracking of short-chain n-paraffins formed as a result of hydrocracking of long-chain n-paraffins, with the production of gasoline hydrocarbons, an increase in its yield and a decrease in the yield of diesel fuel. The most optimal conditions for the process of hydroisodewaxing of the diesel fraction on the 1% Mo/MAS-H-bentonite and the 2% Mo/MAS-H-bentonite were 320 °C, 2 MPa, and 1 h^−1^.

While one compares the performance characteristics of the diesel fraction under optimal process conditions, it can be concluded that of the two samples, the 1% Mo/MAS-H-bentonite catalyst is the most effective, perhaps due to the predominance of easily recoverable octahedral forms of molybdenum according to TPR-H_2_, which are responsible for hydrogenating and dehydrogenating functions. In addition, a decrease in the yield of diesel fraction with an increase in the molybdenum content in the catalyst is probably due to strong acid sites, according to the TPD-NH_3_ data responsible for the hydrocracking process.

The 5% Ni/MAS-H-bentonite showed the best results among the three synthesized catalysts with different nickel contents. With an increase of nickel content from 1 to 2 and further up to 5 wt.% in the catalyst, under optimal process conditions, the yield of diesel fraction was 92.3, 93.1, and 95.4, respectively; this is probably due to the enhancement of the interaction between nickel and support, which is consistent with the data of TPR-H_2_ and IR-Fourier spectroscopy. According to the literature data [51], a nickel content of more than 5 wt.% in the catalyst leads to a decrease in the interaction between the metal and the support due to the agglomeration of an excess nickel phase.

For the studied sample of the bifunctional catalyst containing 5% Ni and 1% Mo, an increase in the process temperature from 260 to 340 °C leads to a decrease in the pour point of the products from −19 to −38 °C. CFPP also decreased from −12 to −36 °C, and the flash point in a closed crucible decreased from 52 to 27 °C. Therefore, the optimal temperature for the process of hydroisodewaxing of the diesel fraction on the studied catalysts was 320 °C—the best performance characteristic with the highest output of diesel fraction of 97.4 wt.% was achieved on bimetallic bifunctional catalysts compared to monometallic ones.

Results of an analysis of the composition of the process products in the presence of 1% Mo/MAS-H-bentonite, 5% Ni/MAS-H-bentonite, and 5% Ni-1% Mo/MAS-H-bentonite are presented in Table 8.

For the bifunctional catalyst 1% Mo/MAS-H-bentonite, the process of hydroisodewaxing proceeds in two directions: hydroisomerization and hydrocracking. An increase in the process temperature from 260 to 340 °C leads to a decrease in the content of higher long-chain paraffinic hydrocarbons; this is due to an increase in the rate of the hydrocracking reaction respectively, a larger amount of paraffin undergoes this reaction. Insignificant changes in the content of naphthenes in the reaction products compared to the feedstock can be explained as follows. In the case of an increase from 32.78 to a maximum of 35.26 wt.%—probably by the fact that monoaromatic hydrocarbons were hydrogenated at high pressure and temperature; in the case of a decrease from 32.78 to at least 24.84 wt.% part of the naphthenes turned into isoparaffins. The low degree of hydrodearomatization of less than 2% may be due to the predominance of strong acid sites over medium acid sites, which play a key role in the hydroisodewaxing process. The decrease in the yield of diesel fraction correlates with the data on the group composition of hydrocarbons and can be explained by an increase in the proportion of hydrocracking.

According to Table 8, on a nickel-promoted catalyst, in contrast to the Mo-containing catalyst, the main reaction of the dewaxing process is the hydroisomerization of higher n-paraffins. The output of isoparaffins, depending on the temperature, pressure, and space velocity of the feedstock, is in a range of values from 16.2 to 29.2 wt.%. Compared to the Mo-containing catalyst, on the 5% Ni/MAS-H-bentonite catalyst, the share of the dearomatization process increases from less than 2 to 3.67%, which may be due to a large number of medium-strength acid sites.

Thus, comparing the best molybdenum-containing catalyst with the best nickel-containing catalyst, it was found that the nickel-promoted catalyst, under identical optimal conditions, showed better performance and greater yield of diesel fraction.

Given the sulfur content in the initial diesel fraction of 251 ppm, nickel catalyst poisoning is possible. Hydrocarbons adsorbed on the Ni-S sites migrate to the aluminum-containing support, where they undergo acid cracking to form cracked products. To solve this problem, the work included a study of the activity of a catalyst with bimetallic systems, i.e., Ni-Mo/MAS-H-bentonite. The role of molybdenum is to attract sulfur to itself and protect nickel from poisoning while nickel itself performs hydrogenation.

Table 8 shows that 5% Ni-1% Mo/MAS-H-bentonite is the most promising catalyst out of the six developed ones as it allows for achieving the highest yield of the diesel fraction of 97.4% due to the hydroisomerization of n-higher paraffins contained in the feedstock. At the same time, low pour point diesel fuel was obtained, which, according to operational characteristics, is close to the winter fuel type.

## 3. Materials and Methods

### 3.1. Bifunctional Catalysts Preparation

One of the acid catalyst supports (mesoporous aluminosilicate) was prepared by co-condensation of tetraethylorthosilicate Si(OC_2_H_5_)_4_ and aluminum sec-butoxide. Hexadecylamine was used as a template. The resulting MAS sample became one of the components of the carrier of the bifunctional catalyst. It was placed in a porcelain bowl together with the pre-activated bentonite from the Tagan field (East Kazakhstan region, Kazakhstan), which was used as a second component of the support and a binding agent in the ratio MAS/H-bentonite—35/65. Activation of bentonite was carried out with 20% sulfuric acid solution. Synthesis of bifunctional mono- and bimetallic catalysts, 1% Ni/MAS-H-bentonite, 2% Ni/MAS-H-bentonite, 5% Ni/MAS-H-bentonite, 1% Mo/MAS-H-bentonite, 2% Mo/MAS-H-bentonite, and 5% Ni-1% Mo/MAS-H-bentonite were prepared using the wet impregnation technique. The procedure of this method and the physicochemical characteristics of the synthesized materials were reported by the authors [21]. All synthesized samples were synthesized on the basis of the Scientific Research Institute for New Chemical Technologies and Materials at the al-Farabi Kazakh National University.

### 3.2. Study of the Physicochemical Characteristics of Bifunctional Catalysts

#### 3.2.1. The Nitrogen Adsorption/Desorption Method

The porous structure of the synthesized samples and the BET surface area were studied using standard nitrogen adsorption/desorption method using Quanta ChromeAutosorb-6 sorbtometer (Quantachrome Instruments, Boynton Beach, FL, USA). Before analysis, the samples were contained at 300 °C for 12 h to a pressure of 3 × 10^−3^ atm. The nitrogen adsorption–desorption isotherm was recorded at a temperature of 77 K. The characteristics of the porous structure were calculated using standard software (NovaWin 10.0 software). The specific surface area was calculated using the BET model (Brunauer-Emmett-Teller) at relative partial pressure P/P_0_ = 0.2. The total pore volume and the distribution of pores by the radiuses were calculated using the BJH model (Barrett-Joyner-Halenda) at relative partial pressure P/P_0_ = 0.95 cm^3^/g.

#### 3.2.2. X-ray Wide-Angle Scattering Methods (XRD)

The ordering of the porous structure of the synthesized materials was analyzed using the method of X-ray wide-angle scattering (XRD). X-ray patterns were taken on a Rigaku D/MAX 2200 diffractometer (Tokyo, Japan) with a Cu K radiation source (λ = 0.15418 nm) at a shooting rate of 1 deg/min.

#### 3.2.3. Diffuse Reflection Infrared Fourier Transform Spectroscopy (DRIFT)

Determination of the Lewis/Brønsted acid sites in the synthesized catalysts was carried out using infrared Fourier transform (DRIFT) experiments of adsorbed pyridine diffuse reflectance using a JASKO FT-IR-4700 spectrometer (Oklahoma, USA). Prior to these tests, the samples were pretreated at 110 °C to remove water adsorbed on the acidic sites. After pyridine adsorption, the samples were dried at 40 °C before DRIFT analysis; the background spectrum was recorded with KBr. The spectrum obtained after pyridine desorption was subtracted from those measured before pyridine adsorption (fresh samples) to determine the bands relative to Lewis and Brønsted acid sites.

#### 3.2.4. Fourier Transform Infrared Spectroscopy (FTIR)

The IR spectrum of the samples was obtained using Fourier transform infrared spectroscopy (FTIR) on a JASCO FTIR-4700 spectrometer with a spectral resolution of 0.4 cm^−1^, using tableting of an aluminosilicate powder with a binder of potassium bromide at a sample to KBr ratio of 3:800.

#### 3.2.5. Temperature-Programmed Desorption of Ammonia (TPD-NH_3_)

Temperature-programmed desorption of ammonia (TPD-NH_3_) was carried out on a USGA-101 unit A 0.1 g sample (fraction 0.30–0.50 mm) was placed in a quartz reactor, calcined at 512 °C in a helium flow (139 min, 20 mL/min), and then cooled. Ammonia adsorption (7 vol.% NH_3_ in N_2_, rate 40 mL/min) was carried out at 102 °C for 1 h. After that, the system was purged with helium (60 min). TPD curves were recorded in a helium flow (8 mL/min) from 60 to 600 °C at a linear temperature rise rate of 8 °C/min.

#### 3.2.6. The Temperature-Programmed Reduction of Hydrogen (TPR-H_2_)

The TPR-H_2_ method will be used to study the reduction of active components on the catalyst surface. Temperature-programmed hydrogen reduction (TPR-H_2_) was carried out on a USGA-101 unit, including a gas preparation system, a flow reactor (internal diameter = 4 mm) with a tubular furnace, and a thermal conductivity detector. A sample (100 mg, fraction 0.30–0.50 mm) was preliminarily purged with Ar at 480 °C for 40 min, followed by cooling to 50 °C and then heated at a rate of 10 °C/min from 50 to 950 °C in a mixture flow of 10 vol.% H_2_ in Ar at a feed rate of 30 cm^3^/min. The gas mixture was analyzed using a thermal conductivity detector.

### 3.3. Study of the Composition and Physicochemical Characteristics of Diesel Fractions before and after Testing

Evaluation of the effectiveness of catalysts in the process of hydroisomerization of diesel fractions includes a comparison of the performance characteristics of the fuel before and after the process. In this regard, the following analyses were carried out: the group hydrocarbon composition of the diesel fraction was determined, sulfur content, flash point in a closed crucible, CFPP, pour point, and fractional composition were determined.

#### 3.3.1. The Chromatography-Mass Spectrometry Method

The group hydrocarbon composition of diesel fractions was studied by chromatography-mass spectrometry on an Agilent 7890A/5975C chromato-mass spectrometer (Santa Clara, CA, USA) with a mass range of 1.6–1050 amu.

#### 3.3.2. Determination of Pour Point of Diesel Fractions

The pour point of diesel fractions before and after the experiment was determined on an automatic apparatus TPZ-LAB-22 in accordance with the standards GOST 20287-91 (method A), ASTM D5950, ASTM D 5771. The parameters of accuracy, convergence, and reproducibility are equivalent and correspond to the results obtained in accordance with the methods ASTM D2500, ASTM D 97, GOST 20287-91 (methods A and B), GOST 5950, EN 23015, ISO 3015, ISO 3016. The apparatus is equipped with a built-in cooling unit capable of cooling the test cell to 95 °C without the use of external cooling systems. The device automatically sets and maintains the temperature of the cooling bath, performs tests, and creates and stores test reports in memory.

#### 3.3.3. The Closed Cup Flash Point Method

The flash point in a closed cup was determined on an apparatus for determining flash point in a closed crucible TVZ-LAB-01 in accordance with GOST 6356-75: Petroleum products. Methods for determining the flash point in a closed crucible.

#### 3.3.4. Method for Determining the CFPP

The CFPP was determined on the analyzer of the quality of gasoline, diesel fuel, and motor oil. “SX-250 octanometer” (SHATOX, Tomsk, Russia) in accordance with GOST 22254-92: Method for determining the CFPP.

#### 3.3.5. Method for Determining the Fractional Composition of the Diesel Fraction

The fractional composition of the starting compounds was determined on the ARN-LAB-1 apparatus for the distillation of petroleum products in accordance with GOST 2177: Petroleum products. Methods for determining fractional composition.

#### 3.3.6. Method for Determining the Sulfur Content in the Diesel Fraction

The mass fraction of sulfur in initial compounds and reaction products was determined on a Spektroscan S X-ray fluorescent energy-dispersive sulfur analyzer (SPECTRON, Saint Petersburg, Russia) in accordance with GOST R 51947: Oil and oil products. Determination of sulfur by energy dispersive X-ray fluorescence spectrometry.

### 3.4. Testing of Catalysts in a Flow-Through Unit

Determination of the catalytic activity of the synthesized catalysts was carried out on a flow-type installation with a fixed catalyst bed in a stream of hydrogen in a temperature range of 240–300 °C; feed rate was 1 h^−1^; pressure 2 MPa. The volume ratio H_2_:eedstock = 100. The authors also reported the scheme of the laboratory catalytic installation and its description [8].

### 3.5. Determination of Errors in Experimental Results

All experiments to study the catalytic activity of the catalysts were carried out three times. Statistical analysis included the calculation of average values (Formula (4)), a squared standard deviation (Formula (5)), and a standard error of the mean value (Formula (6)) [52]:

(1) $$\bar{x}=\frac{\sum_{i=1}^{n}x_{i}}{n}$$

where $\bar{x}$ is a mean value;

*n* is the total number of values $\chi_{i}$;
*x_i_* is the *i*th individual observation.

(2) $$s^{2}=\frac{\sum_{i=1}^{n}(x_{i}-\bar{x}{)}^{2}}{n-1}$$

where *s*^2^ is a squared standard deviation;

$\bar{x}$ is a mean value;
*n* is the total number of values $\chi_{i}$;
*x_i_* is the *i*th individual observation.

(3) $$s_{\bar{x}}=\frac{s}{\sqrt{n}}$$

where $s_{\bar{\chi }}$ is a standard error of the mean value;

*s* is a standard deviation;
$\bar{x}\;$ is a mean value;
*n* is the total number of values $\chi_{i}$;
*x_i_* is the *i*th individual observation.

The standard error of the mean value for all measurements was in the range of 0.1–0.4.

## 4. Conclusions

The influence of the nature and content of promoting additives on the activity of bifunctional catalysts, the acid components of which are mesoporous aluminosilicates and activated bentonite, was studied during the hydrodewaxing of the diesel fraction in a flow-type reactor in the temperature range 260–340 °C, pressure 2 MPa, volumetric feed rate 1 h^−1^. It has been established that the maximum yield of diesel fraction of 97.4% in the process of hydroisodewaxing on a 5% Ni-1% Mo/MAS-H-bentonite catalyst is achieved at a temperature of 320 °C. The resulting diesel fraction meets the requirements for cold-climate fuels: the maximum filterability temperature is minus 33 °C, the flash point in a closed crucible is 39 °C, and the pour point is minus 36 °C.

## Figures and Tables

**Table 1:** Physicochemical properties of the samples.

Catalyst	SSA, m^2^/g	Pore Volume, cm^3^/g	Pore Average Size, nm
MAS-H-bentonite	375	0.3523	3.5
1% Mo/MAS-H-bentonite	284	0.2166	2.17
5% Ni/MAS-H-bentonite	152	0.3565	1.67
5% Ni-1% Mo/MAS-H-bentonite	265	0.2567	3.8

**Table 2:** Concentration and strength of the MAS and MAS-H-bentonite acid sites.

Sample	Amount of the Acid Sites (Amount of Adsorbed NH_3_, µmol/g)			
	Weak T_max_ <300 °C	Medium T_max_ = 300–500 °C	Strong T_max_ >500 °C	Total
MAS	1144	27	-	1171
MAS-H-bentonite	1193	-	-	1193

**Table 3:** Concentration and strength of acid sites of the synthesized bifunctional catalysts based on mesoporous aluminosilicates and pre-activated bentonite.

Sample	Amount of the Acid Sites (Amount of Adsorbed NH_3_, µmol/g)			
	Weak T_max_ < 300 °C	Medium T_max_ = 300–500 °C	Strong T_max_ > 500 °C	Total
1% Mo/MAS-H-bentonite	419	81	120	620
2% Mo/MAS-H-bentonite	440	116	128	684
1% Ni/MAS-H-bentonite	255	162	29	446
2% Ni/MAS-H-bentonite	252	174	6	432
5% Ni/MAS-H-bentonite	231	196	94	521
5% Ni-1% Mo/MAS-H-bentonite	491	122	130	743

**Table 4:** Data from the temperature-programmed hydrogen reduction of bifunctional catalysts.

Sample	Temperature, °C	Amounts of H_2_ Consumed, µmol/g
1% Mo/MAS-H-bentonite	544	170
	658	15
	688	14
	814	1
2% Mo/MAS-H-bentonite	589	651
	834	142
1% Ni/MAS-H-bentonite	328	127
	472	144
	532	37
	683	126
	792	2
	840	33
	858	57
2% Ni/MAS-H-bentonite	415	127
	603	930
	710	81
	763	13
	866	2
5% Ni/MAS-H-bentonite	411	124
	571	164
	657	4
5% Ni-1% Mo/MAS- H-bentonite	317	29
	460	670
	529	406
	702	472
	792	41
	827	15

**Table 5:** Characteristics of the diesel fraction.

Indicators	Values
Sulfur content, ppm	251
Flashpoint in a closed cup, °C	66
CFPP, °C	−10
Pour point, °C	−12
Flow point, °C	−5
Fractional composition:	
Boiling point, °C	197
Fraction yields up to a temperature of 350 °C, % vol.	97

**Table 6:** Group hydrocarbon composition of the diesel fraction.

Compounds	Content, wt.%
N-paraffins	18.15
Iso-paraffins	16.08
Naphthenes	32.78
Aromatic hydrocarbons, %	26.31
From them:	
monocyclic	24.3
polycyclic	2.01
Olefins	1.72
Heteroatomic compounds	4.96
Total	100.0

**Table 7:** Performance characteristics and yield of diesel fraction after hydroisodewaxing process.

Catalyst	T, °C	CFPP, °C	Flash Point in a Closed Cup, °C	Pour Point, °C	Diesel Fraction Yield, %
1% Mo/MAS-H-bentonite	260	−14	52	−26	93.6
	280	−19	50	−31	90.6
	300	−27	45	−36	87.5
	320	−38	36	−38	85.2
	340	−42	20	−41	72.4
2% Mo/MAS-H-bentonite	260	−16	51	−27	92.8
	280	−18	47	−28	89.2
	300	−27	43	−31	85.7
	320	−33	35	−36	84.0
	340	−36	31	−40	73.2
1% Ni/MAS-H-bentonite	260	−12	53	−19	97.0
	280	−16	50	−21	96.7
	300	−23	47	−23	94.8
	320	−27	41	−26	92.3
	340	−30	37	−29	90.4
2% Ni/MAS-H-bentonite	260	−12	55	−18	97.0
	280	−15	52	−19	95.9
	300	−21	48	−22	94.3
	320	−25	43	−24	93.1
	340	−28	38	−27	90.0
5% Ni/MAS-H-bentonite	260	−15	53	−20	97.0
	280	−17	50	−21	96.5
	300	−26	46	−25	96.3
	320	−31	41	−30	95.4
	340	−35	33	−33	93.3
5% Ni-1% Mo/ MAS- H-bentonite	260	−12	52	−19	97.2
	280	−16	50	−22	97.5
	300	−22	45	−27	96.3
	320	−33	39	−36	97.4
	340	−36	27	−38	96.9

**Table 8:** Comparative table of indicators of raw materials and reaction products obtained on 1% Mo/MAS-H-bentonite, 5% Ni/MAS-H-bentonite, and 5% Ni-1% Mo/MAS-H-bentonite catalysts.

Indicators	Raw Material	Reaction Products Obtained on Catalysts at 320 °C, 2 MPa, 1 h^−1^		
		1% Mo/MAS-H-Bentonite	5% Ni/MAS- H-Bentonite	5% Ni-1% Mo/ MAS- H-Bentonite
The content of n-paraffins C_10_-C_27_, wt.%	18.15	9.6	12.6	10.6
The content of iso-paraffins, wt.%	16.08	24.3	22.4	25.3
Naphthenes	32.78	33.01	35.16	33.04
Aromatic	26.31	25.85	23.81	25.16
hydrocarbons, %				
From them:				
monocyclic	24.3	24.7	23.64	24.26
polycyclic	2.01	1.15	0.17	0.90
Hydrocracking products, wt.%	-	8.5	0.7	1.2

## Data Availability

Not applicable.
